# Effects and safety of *Ginkgo biloba* on depression: a systematic review and meta-analysis

**DOI:** 10.3389/fphar.2024.1364030

**Published:** 2024-03-18

**Authors:** Jingya Lin, Xiaojing Sun, Lingli Yang

**Affiliations:** Department of Neurology, TongRen Hospital, Shanghai Jiao Tong University School of Medicine, Shanghai, China

**Keywords:** *Ginkgo biloba*, GKB, depression, depressive symptoms, meta-analysis

## Abstract

**Background:** Because depression is a major factor contributing to the global disease burden, we tried to analyze the effects and safety of *Ginkgo biloba* (GKB) on patients with depression.

**Methods:** We conducted a literature search for articles published between January 2002 and May 2022 in seven online databases (PubMed, Scopus, Embase, Google Scholar, Web of Sciences, Cochrane Library, and China National Knowledge Infrastructure). A systematic literature review and meta-analysis were performed to compare the effects and safety of GKB on patients with depression, including subjective and objective indicators of depression evaluation.

**Results:** In total, 21 eligible articles with nine indicators among 2074 patients were included. Several outcomes showed a difference, and the GKB group had better results than the control group, including the Hamilton Depression Scale (HAMD), after taking GKB for 4 weeks (MD = −2.86, 95%CI [−4.27, −1.46], *p* < 0.01), 6 weeks (mean difference (MD) = −3.36, 95%CI [−4.05, −2.67], *p* < 0.01), and 8 weeks (MD = −4.58, 95% CI [−6.11, −3.05], *p* < 0.01), modified Barthel index (MBI) (MD = 14.86, 95%CI [12.07, 17.64], *p* < 0.01), modified Edinburgh-Scandinavian stroke scale (MESSS) (MD = −4.57, 95%CI [−6.34, −2.79], *p* < 0.01), brain-derived neurotrophic factor (BDNF) (MD = 16.35, 95%CI [7.34, 25.36], *p* < 0.01), 5-hydroxytryptamine (5-HT) (MD = 4.57, 95%CI [3.08, 6.05], *p* < 0.01), and clinical efficacy (risk ratio, RR = 1.24, 95%CI [1.17, 1.32], *p* < 0.01). However, there were no differences in adverse events between GKB and controls.

**Conclusion:** In conclusion, the main finding was that patients treated with GKB had better MBI, MESSS, BDNF, 5-HT, and HAMD values after 4 weeks, 6 weeks, and 8 weeks than the control group. GKB might reduce the risk of depression or depressive symptoms with safe clinical efficacy.

**Systematic Review Registration:** identifier (INPLASY2023100052)

## Introduction

It is reported that a main factor contributing to the burden of disease throughout the world is mental illness (Liu et al., 2020). The two most incapacitating mental diseases, depression and anxiety disorders, are among the top 25 major causes of global disease burden in 2019, according to Global Burden of Disease (GBD) research (Collaborators, 2021). Of these, depression is a severe mood disorder characterized by a dearth of pleasure, diminished capacity for pleasure, sleepiness or insomnia, psychomotor agitation or retardation, exhaustion, feelings of worthlessness or guilt, trouble concentrating, and recurrent thoughts of suicide or death (Yuehua Li, 2006; Wang, 2010). According to WHO, depression is the presence of persistent sadness and the loss of interest in activities that one normally enjoys, accompanied by an inability to carry out daily activities for at least 2 weeks. It is different from the usual mood fluctuations or temporary sadness in response to challenges in everyday life (Collaborators, 2021).

After the COVID-19 pandemic, respiratory-transmitted illnesses continue to generate concern about the implications on mental health due to their immediate psychological effects and long-term economic and societal repercussions (Collaborators, 2021). Globally, economic activity is only slowly recovering, unemployment rates are rising, employment rates for young people—especially college students—are falling, and there are still widespread epidemics of respiratory diseases (Yueyi Kan and Rao, 2022). All factors have the potential to have a negative impact on people’s mental health. We must continue to identify effective ways to lessen the negative effects of the final phase of the COVID-19 pandemic on mental health, and we still need up-to-date data on the incidence and burden of mental illnesses across the world (Zhang et al., 2022).

There is evidence that ginkgo has favorable benefits in older persons with Alzheimer’s disease, multi-infarct dementia, and moderate cognitive impairment (Woelk et al., 2007; Dou, 2013). Ginkgo is used to treat cerebral insufficiency and intermittent claudication. Ginkgo biloba extract EGb761 has been utilized extensively in the treatment of central nervous system conditions such as age-related cognitive decline and dementia, vestibular and non-vestibular vertigo, tinnitus, and peripheral artery occlusive disease (Fournier et al., 2010; Yeung et al., 2018). Because EGb 761 is able to modify blood rheology to enhance blood flow as well as to promote neuroprotection and modulate neurotransmission, the mechanism of action behind these therapeutic benefits is complex (Kasper, 2015). Additionally, it has been discovered that people with mental impairment who take EGb 761 have less anxiety. Consequently, it is of significant clinical relevance to evaluate the efficacy and safety of GKB on mental health.

There is still some debate about evaluating the use of ginkgo for depression treatment because there is not enough research on the herb, and not all reports agree that it works well. In order to undertake a systematic analysis with high confidence, the current study attempted to incorporate all trials on ginkgo for depression conducted globally over a 20-year period. The main focus is to evaluate if GKB is effective and safe for depression, and we assume GKB is effective in ameliorating depression.

## Methods

### Research setup

Based on the PICO framework, we selected people with depression as patients. The intervention was GKB, and it was compared with placebo or traditional antipsychotics. The outcome included the Hamilton Depression Scale (HAMD), the modified Barthel index (MBI), the modified Edinburgh-Scandinavian stroke scale (MESSS), brain-derived neurotrophic factor (BDNF), 5-hydroxytryptamine (5-HT), and adverse events.

### Literature search strategy

The Preferred Reporting Items for Systematic reviews and Meta-Analyses (PRISMA) flow diagram and checklist (Page et al., 2021), which were registered at the International Platform of Registered Systematic Review and Meta-analysis Protocols (INPLASY2023100052), served as the foundation for this study. To find all randomized controlled trials evaluating GKB for depression, we searched seven internet databases (PubMed, Embase, Scopus, Web of Sciences, Google Scholar, Cochrane Library, and China National Knowledge Infrastructure) for pertinent articles. After a thorough screening, 21 publications, released without language restriction between January 2002 and May 2023, were included.

In the Boolean phrase ((Ginkgo biloba) OR (G. biloba) OR GKB) AND ((depression) OR (depressive symptoms)), the medical topic terms and keywords utilized for the search were “Ginkgo biloba,” “G. biloba,” “GKB,” “depression,” and “Depressive Symptoms.” The references’ related articles were also investigated. Studies qualified if they included the following data: observational studies reporting clinical effectiveness, adverse events, and random control trials reporting depression assessments. Evaluation of GKB’s impact on depression was the main goal.

Two authors (Jingya Lin Xiaojing Sun) extracted the data using a standardized data form after screening the titles, abstracts, and full texts of the publications identified by our search and assessing the risk of bias. Any discrepancies were discussed with a third author (Lingli Yang).

### Inclusion and exclusion criteria

After the initial selection of the studies, the pertinent texts were examined, and the studies needed to fulfill the following criteria:

(1) Studies that compared GKB and a control for the treatment of depression;
(2) The design of research should be randomized control trials;
(3) Containing indicators evaluating the efficacy and safety of GKB on depression;
(4) Available in full text.

The following predetermined exclusion criteria were used to disqualify studies from consideration:

(1) Research on other drugs;
(2) Other topics about depression;
(3) Study lacking available data;
(4) Review, abstract, or duplicate publication.

### Data extraction and quality assessment

Two writers (Jingya Lin Xiaojing Sun) extracted the following information from each included study: the name of the first author, publication year, author’s country, groups based on intervention (GKB and control), research design, gender, average subject age, and research cycle. The outcome criteria for depression were GKB’s effectiveness and safety.

Using the ROB 2.0 scale (a revised Cochrane risk-of-bias instrument for randomized trials), two independent reviewers (Xiaojing Sun and Lingli Yang) assessed the caliber of the included studies.

### Statistical analysis

We assessed heterogeneity using the Cochrane Q-statistic and Higgins and Thompson’s I^2^. Depending on its value, I^2^ was used to classify heterogeneity as low, moderate, or high: 25%, 50%, or 75%. When I^2^ was less than 50%, the fixed-effect model was used for the meta-analyses. If I^2^ was higher than 50%, the random-effect model was used in all other cases. For effect sizes of continuous outcomes, the mean difference (MD) with 95% CI (confidential interval) was precisely recalculated. Risk ratios (RRs) were computed for categorical outcomes. Our statistical analyses were conducted using R software version 4.2.1 (R Core Team, Vienna, Austria) and the R package meta (version 6.2.0). Statistical significance was defined as a *p*-value of 0.05 or less.

Publication bias was assessed for the Hamilton Depression Scale (HAMD) as the primary outcome using Egger’s regression test and a funnel plot. We ran a sensitivity analysis, deleting one included article at a time, to assess the robustness of the final results.

## Results

### Search process

**FIGURE 1:**
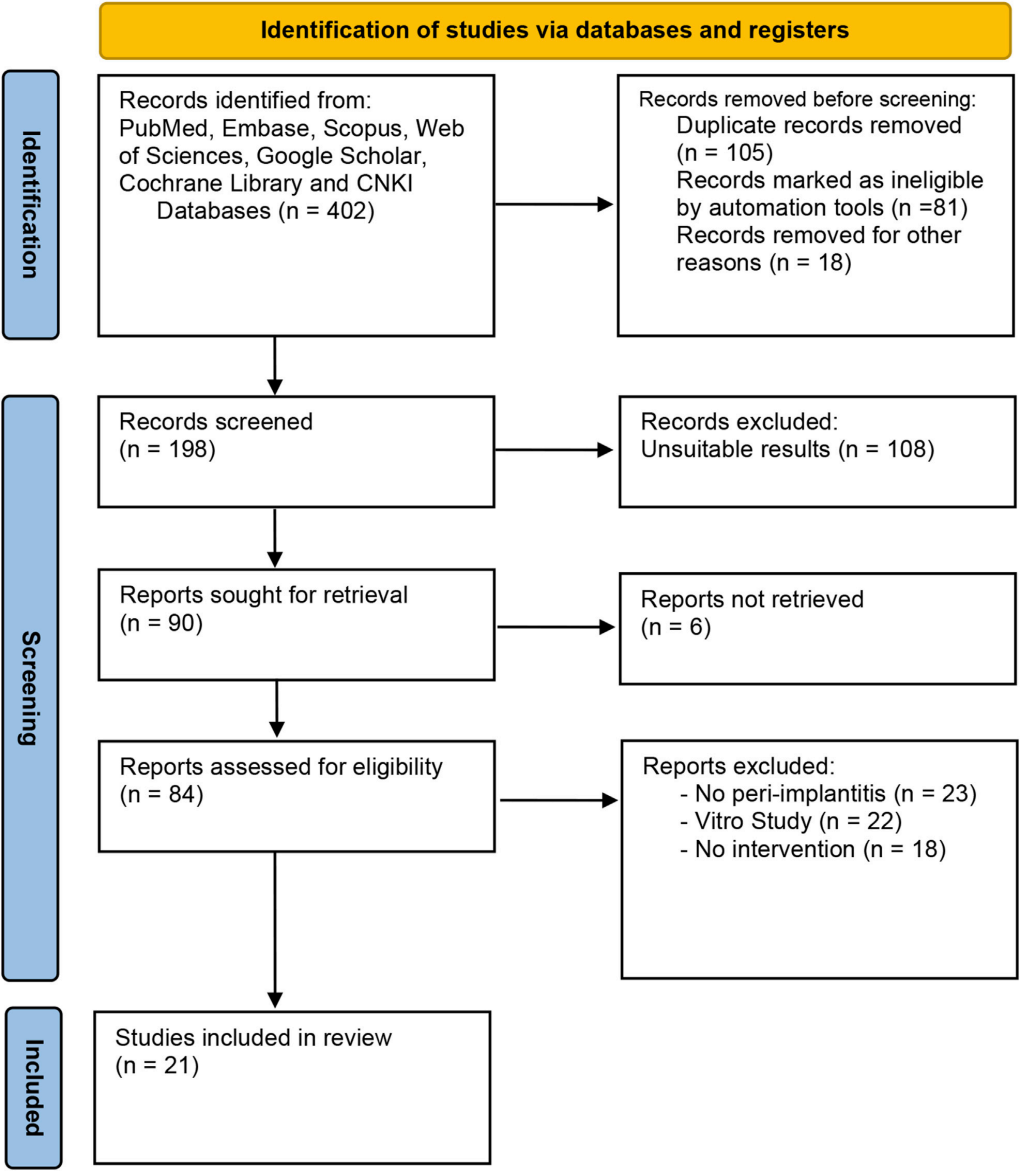
PRISMA diagram of data selection.

The systematic search of seven online databases identified 402 articles based on the PRISMA flow diagram and inclusion/exclusion criteria. Twenty-one publications (Lin, 2002; Fengqing Yu and Liu, 2004; Hartley et al., 2004; Kefeng Guo and Yan, 2006; Wei Chen, 2006; He, 2007; Binhua Chen and Su, 2011; Jia Yuan and Gan, 2011; Xiangdong Luo and Zhou, 2011; Xiangdong Luo and Zhou, 2012; Zhang, 2012; Gavrilova et al., 2014; Hao Song and Chen, 2014; Haixia Wu and Hao, 2016; Shichao and Chen, 2017; Dai et al., 2018; Ru Liu and Zhang, 2018; Junfeng Yin, 2019; Liang et al., 2019; Liu, 2020; Aijun Zhang and Yu, 2021) with 2074 patients were qualified for this meta-analysis after duplicate studies were excluded and eligible studies were screened [12–24]. The research selection flowchart is depicted in Figure 1, and the characteristics of the studies are listed in Table 1. The risk-of-bias evaluation for every included study is displayed in Supplementary Figures S1, S2. In addition, we list the chemical component or manufacturer of the GKB in Table 2 so we can make the evaluation of GKB clear.

**TABLE 1:** Main characteristics of the included studies.

Study	Study	Year	Language	Country	Group	Intervention details	No. of patients (male/female)	Age range (mean)	n	Study design	Study date range
Binhua Chen	Chen	2011	Chinese	China	Intervention with GKB	*Ginkgo biloba* extract (40 mg/capsule)+Citalopram (20 mg/capsule) (1.36 ± 0.45 g/day)	18/14	65.36 ± 5.27	32	RCT	January 2008 through June 2010
					Control	Citalopram (20 mg/capsule)	16/14	63.53 ± 6.17	30		
Wei Chen	Chen	2006	Chinese	China	Intervention with GKB	Ginkgo leaf extract + Prozac	-	-	46	RCT	March 2002 through November 2004
					Control	Prozac	-	-	32		
Chunxiao Dai	Dai	2018	English	China	Intervention with GKB	*Ginkgo biloba* tablets + citalopram	33/35	66.48 ± 4.12	68	RCT	March 2015 through March 2017
					Control	Citalopram	31/37	66.82 ± 3.35	68		
Gavrilova	Gavrilova	2014	English	Russia	Intervention with GKB	*Ginkgo biloba* extract	22/58	65 ± 7	80	RCT	-
					Control	Placebo	13/66	63 ± 7	79		
Kefeng Guo	Guo	2006	Chinese	China	Intervention with GKB	*Ginkgo biloba* extract (19.2 mg/capsule) + paroxetine (20 mg/capsule) (1.36 ± 0.45 g/day)	17/23	-	40	RCT	January 2003 through April 2004
					Control	Paroxetine (20 mg/capsule)	16/22	-	38		
Hartley	Hartley	2004	English	United Kingdom	Intervention with GKB	Gincosan (120 mg *Ginkgo biloba*)	-	58.4 ± 1.0	30	RCT	-
					Control	Placebo	-	57.4 ± 0.7	27		
Jianzhong He	He	2007	Chinese	China	Intervention with GKB	Ginkgo leaf extract	22/23	67.1 ± 2.5	45	RCT	-
					Control	Placebo	24/21	68.1 ± 23	45		
Shichao Li	Li	2017	Chinese	China	Intervention with GKB	Ginkgo leaf extract	30/20	53.3 ± 1.8	50	RCT	January 2015 through January 2016
					Control	Placebo	29/21	52.1 ± 1.6	50		
Zihong Liang	Liang	2019	English	China	Intervention with GKB	*Ginkgo biloba* extract + venlafaxine	22/18	60.86 ± 8.63	40	RCT	-
					Control	Venlafaxine	19/54	60.52 ± 8.68	40		
Chuan Lin	Lin	2022	Chinese	China	Intervention with GKB	*Ginkgo biloba* tablets + nimodipine	26/16	57.2 ± 2.2	42	RCT	November 19 through April 2001
					Controls	Nimodipine	19/17	58.1 ± 3.2	36		
Ru Liu	Liu	2018	Chinese	China	Intervention with GKB	*Ginkgo biloba* tablets + butylphthalide	27/32	68.2 ± 5.6	59	RCT	March 2016 through August 2017
					Control	Butylphthalide	28/31	68 ± 5.5	59		
Yajuan Liu	Liu	2020	Chinese	China	Intervention with GKB	*Ginkgo biloba* tablets + metformin	27/24	63.4 ± 13.72	51	RCT	January 2018 and through January 2019
					Control	Metformin	28/23	64.11 ± 1.38	51		
Xiangdong Luo	Luo	2011	Chinese	China	Intervention with GKB	*Ginkgo biloba* extract (80 mg/capsule) + paroxetine (20 mg/capsule) (1.36 ± 0.45 g/day)	22/25	69.37 ± 2.24	47	RCT	February 2008 through February 2011
					Control	Paroxetine (20 mg/capsule)	19/24	67.45 ± 4.19	43		
Xiangdong Luo	Luo	2012	Chinese	China	Intervention with GKB	*Ginkgo biloba* extract (80 mg/capsule) + paroxetine (20 mg/capsule) (1.36 ± 0.45 g/day)	22/25	69.37 ± 2.24	47	RCT	February 2008 through February 2011
					Control	Paroxetine (20 mg/capsule)	19/24	67.45 ± 4.19	43		
Hao Song	Song	2014	Chinese	China	Intervention with GKB	Ginkgo leaf extract	29/28	51.95 ± 9.04	57	RCT	November 2010 through November 2013
					Control	Placebo	26/22	52.08 ± 8.18	48		
Haixia Wu	Wu	2016	Chinese	China	Intervention with GKB	Ginkgo leaf extract (20 mg/capsule) + fluoxetine (20 mg/capsule)	34/31	64.3 ± 3.4	65	RCT	May 2014 through March 2015
					Control	Fluoxetine (20 mg/capsule)	37/28	64.1 ± 3	65		
Junfeng Yin	Yin	2019	Chinese	China	Intervention with GKB	*Ginkgo biloba* tablets + sertraline	27/23	68.93 ± 9.24	50	RCT	February 2016 through February 2018
					Control	Sertraline	26/24	67.29 ± 8.42	50		
Fengqing Yu	Yu	2004	Chinese	China	Intervention with GKB	Ginkgo leaf extract	14/12	62 ± 5	26	RCT	May 2001 through May 2003
					Control	Placebo	10/11	60 ± 7	21		
Jia Yuan	Yuan	2011	Chinese	China	Intervention with GKB	Ginkgo leaf extract	56/40	-	96	RCT	February 2007 and May 2010
					Control	Placebo	50/44	-	94		
Yujun Zhang	Zhang	2012	Chinese	China	Intervention with GKB	Ginkgo leaf extract	23/29	67 ± 4	52	RCT	January 2007 through June 2011
					Control	Placebo	25/27	68 ± 5	52		
Aijun Zhang	Zhang	2021	Chinese	China	Intervention with GKB	*Ginkgo biloba* extract (80 mg/capsule) + paroxetine (20 mg/capsule) (1.36 ± 0.45 g/day)	22/18	67.54 ± 7.85	40	RCT	March 2018 through January 2019
					Control	Paroxetine (20 mg/capsule)	24/16	65.26 ± 6.34	40		

**TABLE 2:** Chemical component of the included studies.

Study	Year	Country	GKB chemical component
Chen	2011	China	There is no chemical component, but we report the manufacturer: EGb Tablets (produced by Zhejiang Kangenbei Pharmaceutical Co., Ltd.)
Chen	2006	China	Not available
Dai	2018	China	There is no chemical component, but we report the manufacturer: EGb Tablets (Harbin HaoBo Pharmaceutical Co., Ltd.).
Gavrilova	2014	Russia	EGb 761 is a dry extract from *G. biloba* leaves (35–67:1); extraction solvent: acetone 60% (w/w). The extract is adjusted to 22.0%–27.0% ginkgo flavonoids calculated as ginkgo flavone glycosides and 5.0%–7.0% terpene lactones consisting of 2.8%–3.4% ginkgolides A, B, and C and 2.6%–3.2% bilobalide and contains less than 5 ppm ginkgolic acids
Guo	2006	China	There is no chemical component, but we report the manufacturer: Ginkgo Leaf (produced by Guizhou Xinbang Pharmaceutical Co., Ltd., Chinese medicine: Z20028023)
Hartley	2004	United Kingdom	Gincosan capsules (Pharmaton SA, Switzerland). These capsules contain the standard ginkgo extract GK501 (Pharmaton SA, Switzerland) and the ginseng extract G115 (Pharmaton SA, Switzerland)
He	2007	China	Not available
Li	2017	China	There is no chemical component, but we report the manufacturer: *Ginkgo biloba* (approval number: national medicine standard H14023515, Shanxi Pude Pharmaceutical Co., Ltd.)
Liang	2019	China	Not available
Lin	2022	China	There is no chemical component, but we report the manufacturer: GBE761 produced by German Willmar Schwabe Pharmaceutical Co., Ltd.
Liu	2018	China	There is no chemical component, but we report the manufacturer: *Ginkgo biloba* capsule (Chinese medicine: Z20040100)
Liu	2020	China	There is no chemical component, but we report the manufacturer: Ginkgo folic acid capsule (Beijing Maidihai Pharmaceutical Co., Ltd., Chinese medicine: H11020317)
Luo	2011	China	There is no chemical component, but we report the manufacturer: Ginkgo biloba leaves (trade name: Danakang, produced by Bofu-Ipson Company)
Luo	2012	China	There is no chemical component, but we report the manufacturer: *Ginkgo biloba* leaves (trade name: Danakang, produced by Bofu-Ipson Company)
Song	2014	China	There is no chemical component, but we report the manufacturer: *Ginkgo biloba* capsule (Hunan Hansen Pharmaceutical Co., Ltd., batch number: 20100819)
Wu	2016	China	There is no chemical component, but we report the manufacturer: Ginkgo biloba extract (Chinese medicine Z20055358)
Yin	2019	China	Not available
Yu	2004	China	There is no chemical component, but we report the manufacturer: *Ginkgo biloba* leaves produced by Shanghai Xingling Pharmaceutical Factory
Yuan	2011	China	Not available
Zhang	2012	China	There is no chemical component, but we report the manufacturer: *Ginkgo biloba* (produced by Shenzhou Pharmaceutical Industry)
Zhang	2021	China	Ginkgo honey ring oral liquid (national medicine standard: H20013079, produced by Qionglai Tianyin Pharmaceutical Co., Ltd.)

Based on the ROB 2.0, it was determined that the overall quality of the included studies was sufficient in terms of selection bias, comparability quality, and outcome quality.

### Characteristics of included studies

Of all the studies, 10 had more than 100 patients, while 11 had less than 100. The duration of the study varied from 1 to 4 years. All the research designs used in the included articles were randomized control trials (RCTs). The median ages of the patients studied in the included articles ranged from 51.95 to 69.37. There were nearly equal numbers of men and women (952 men and 1,020 women). The specific traits of the included articles are displayed in Table 1.

### Results of quality assessment

The Supplementary Figures S1, S2 show the ROB 2.0 summary and quality assessment details, respectively. More than 50% of the articles indicated a low risk of bias, while less than 20% exhibited a high risk. Only three articles had a high overall bias risk, six had a medium bias risk, and twelve had a low bias risk.

### Results of the heterogeneity test

**FIGURE 2:**
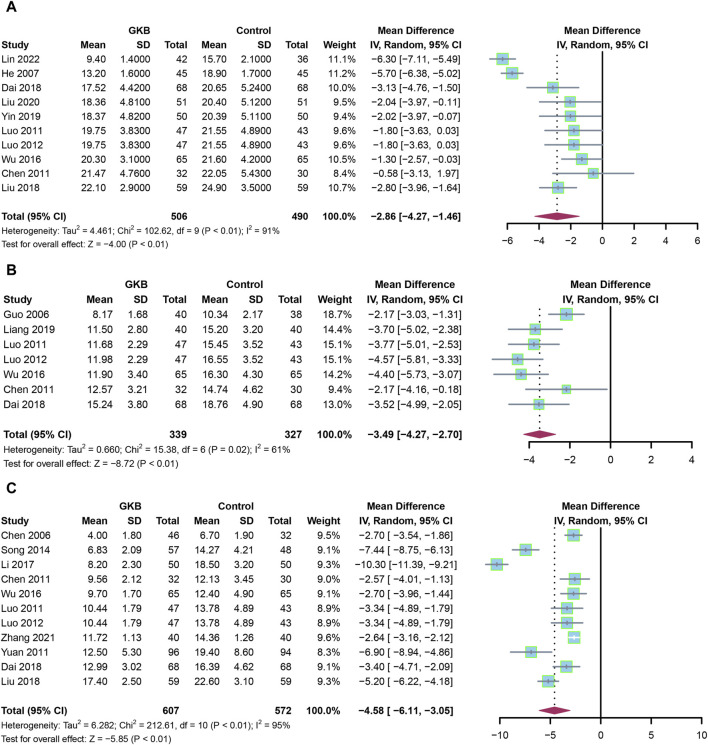
HAMD scales of GKB on patients with depression after taking GKB: **(A)** HAMD scale after taking GKB for 4 weeks. **(B)** HAMD scale after taking GKB for 6 weeks. **(C)** HAMD scale after taking GKB for 8 weeks. Abbreviations: SD: standard deviation; 95% CI: 95% confidence interval; Chi^2^: chi-squared test; Tau2: tau-squared; I^2^: I-squared; P: probability.

First, we evaluated the HAMD scale after giving GKB for 4 weeks, 6 weeks, and 8 weeks. Pooled estimates from 10 studies showed that patients receiving GKB had substantially lower 4-week HAMD scores than the control group (MD = −2.86, 95%CI [−4.27, −1.46], *p* < 0.01, I^2^ = 91%) (Figure 2A). The 6-week HAMD score was documented in seven studies. The results showed that patients receiving GKB also had lower 6-week HAMD scores than the control group (MD = −3.36, 95%CI [−4.05, −2.67], *p* < 0.01, I^2^ = 50%) (Figure 2B). After taking GKB for 8 weeks, the patients receiving GKB had lower HAMD scores than the control group, according to a meta-analysis of 11 publications (MD = −4.58, 95% CI [−6.11, −3.05], *p* < 0.01, I^2^ = 95%) (Figure 2C).

**FIGURE 3:**
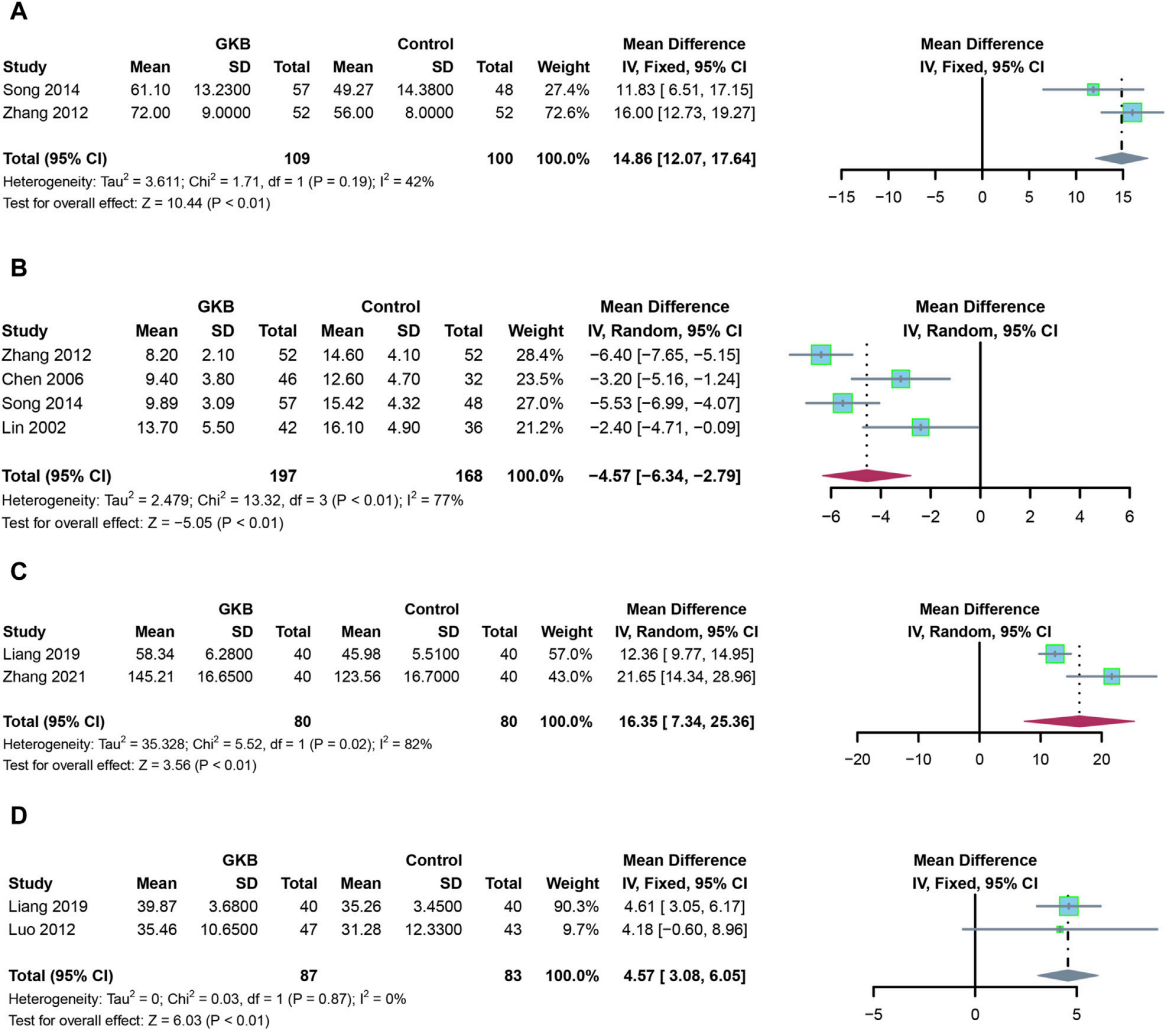
Effects of GKB on patients with depression, including other scales and serum indexes: **(A)** MBI, **(B)** MESSS, **(C)** 5-HT, and **(D)** BDNF. Abbreviations: SD: standard deviation; 95% CI: 95% confidence interval; Chi^2^: chi-squared test; Tau2: tau-squared; I^2^: I-squared; P: probability.

In addition to GKB therapy, we further investigate the serum indices and other depression scales. Patients receiving GKB had a higher MBI than those in the control group (Figure 3A, MD = 14.86, 95%CI [12.07, 17.64], *p* < 0.01, I^2^ = 42%). Meanwhile, patients receiving GKB had lower MESSS values than those in the control groups, according to a pooled analysis (Figure 3B, MD = −4.57, 95%CI [−6.34, −2.79], *p* < 0.01, I^2^ = 77%). Serum levels of BDNF (MD = 16.35, 95%CI [7.34, 25.36], *p* < 0.01, I^2^ = 82%, Figure 3D), and 5-HT revealed that patients receiving GKB had greater values than those in the control groups (MD = 4.57, 95%CI [3.08, 6.05], *p* < 0.01, I^2^ = 0%, Figure 3C).

**FIGURE 4:**
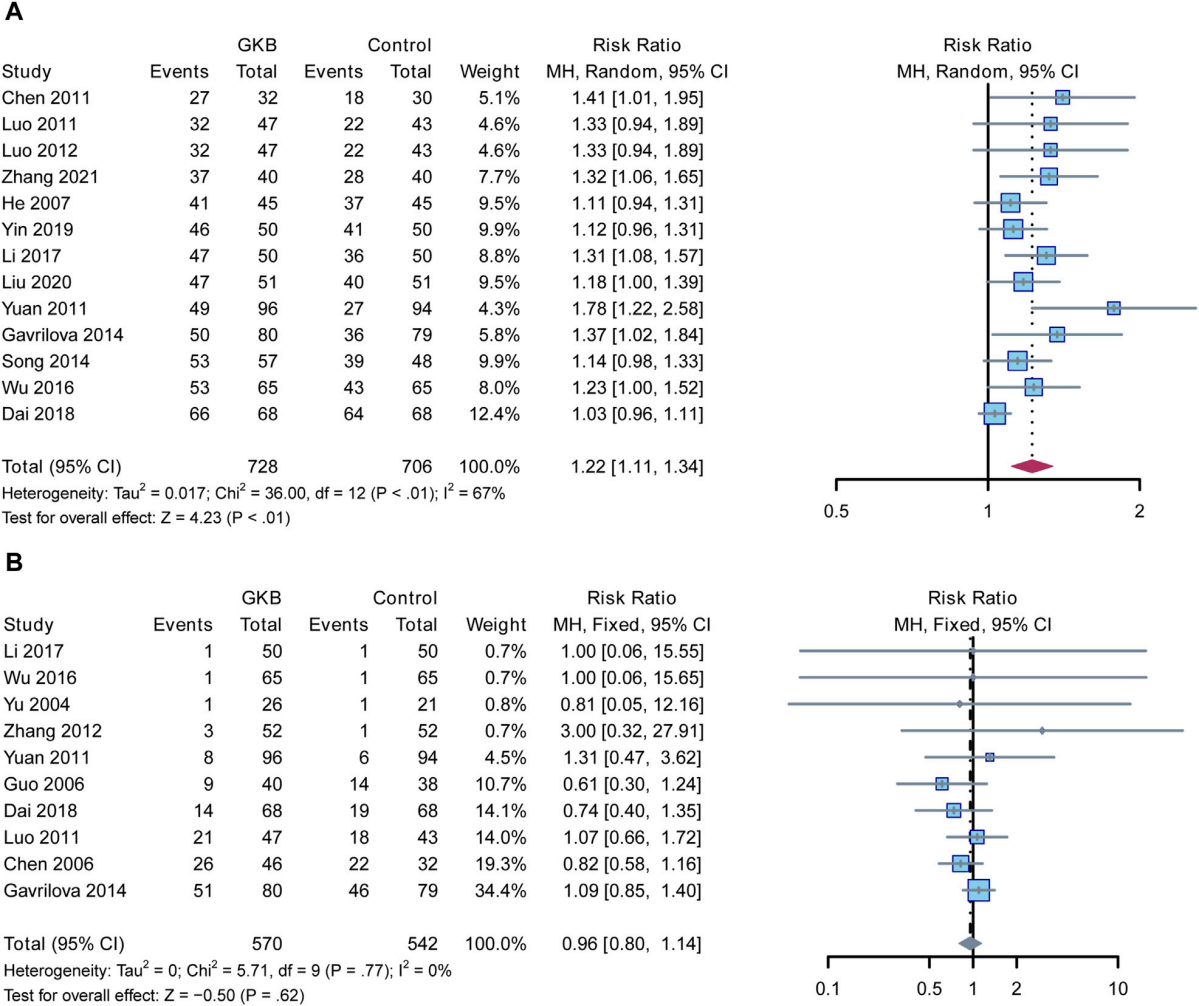
linical efficacy and adverse events reported about the effects of GKB on patients with depression: **(A)** Clinical efficacy and **(B)** Adverse events. Abbreviations: 95% CI: 95% confidence interval; Chi^2^: chi-squared test; Tau2: tau-squared; I^2^: I-squared; P: probability.

Finally, we assessed the clinical effectiveness and adverse events in the GKB and control groups. In a meta-analysis of clinical efficacy, the GKD groups outperformed the control groups in terms of clinical efficacy (RR = 1.22, 95%CI [1.11, 1.34], *p* < 0.01, I^2^ = 67%) (Figure 4A), whereas there was no difference between the GKB and the control group in terms of adverse events (RR = 0.96, 95%CI [0.8, 1.14], *p* = 0.62, I^2^ = 0%) (Figure 4B).

### Results of sensitivity analysis and publication bias

**FIGURE 5:**
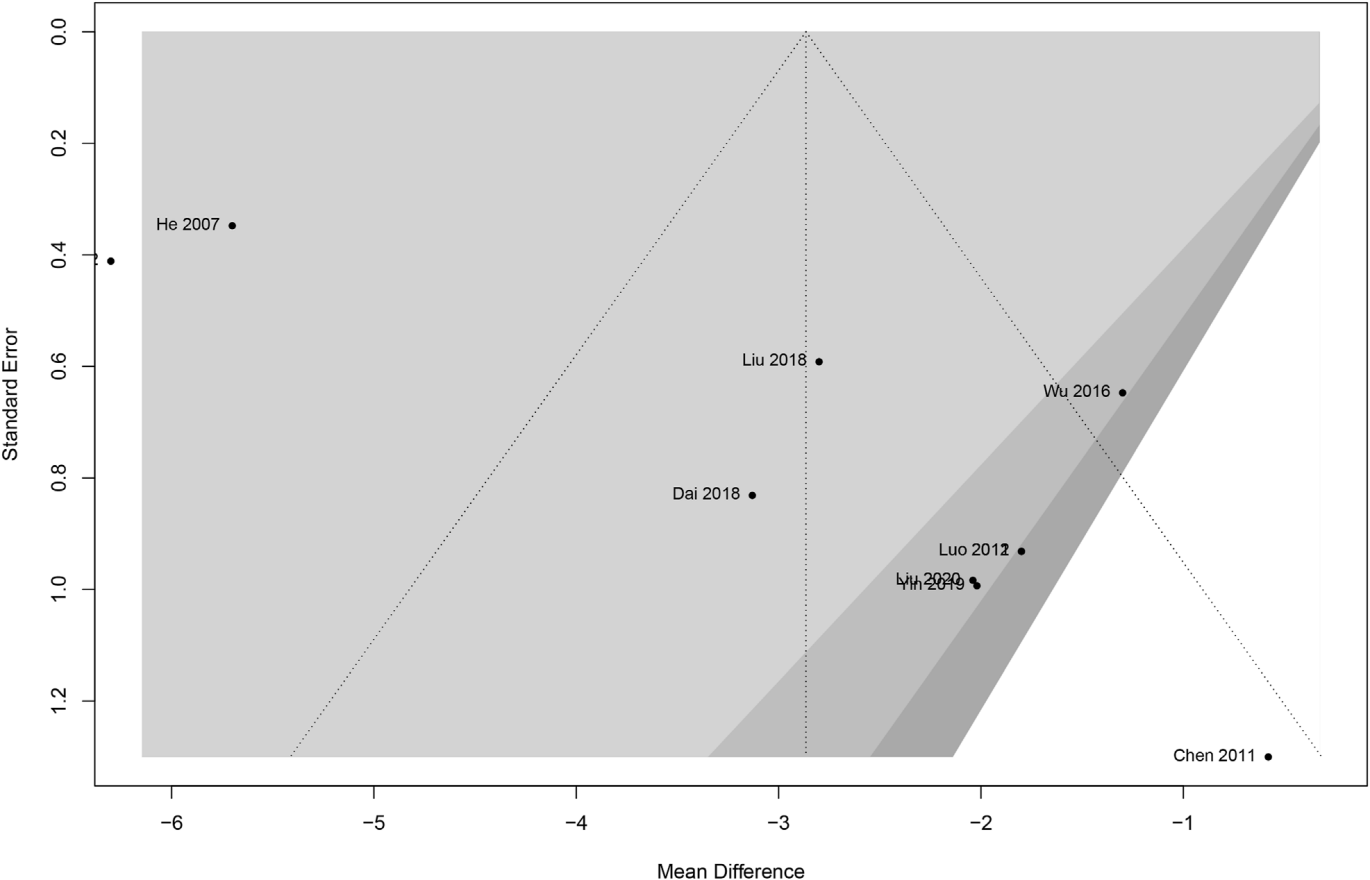
Funnel plot for potential publication bias.

Begg funnel plots for the meta-analysis of adverse events were created to examine the publication bias. The funnel plots (Figure 5) demonstrated visual symmetry and revealed a low level of publication bias in this study. The Egger regression test for adverse events further indicated there was no publication bias (z = 0.23; *p* = 0.19).

**FIGURE 6:**
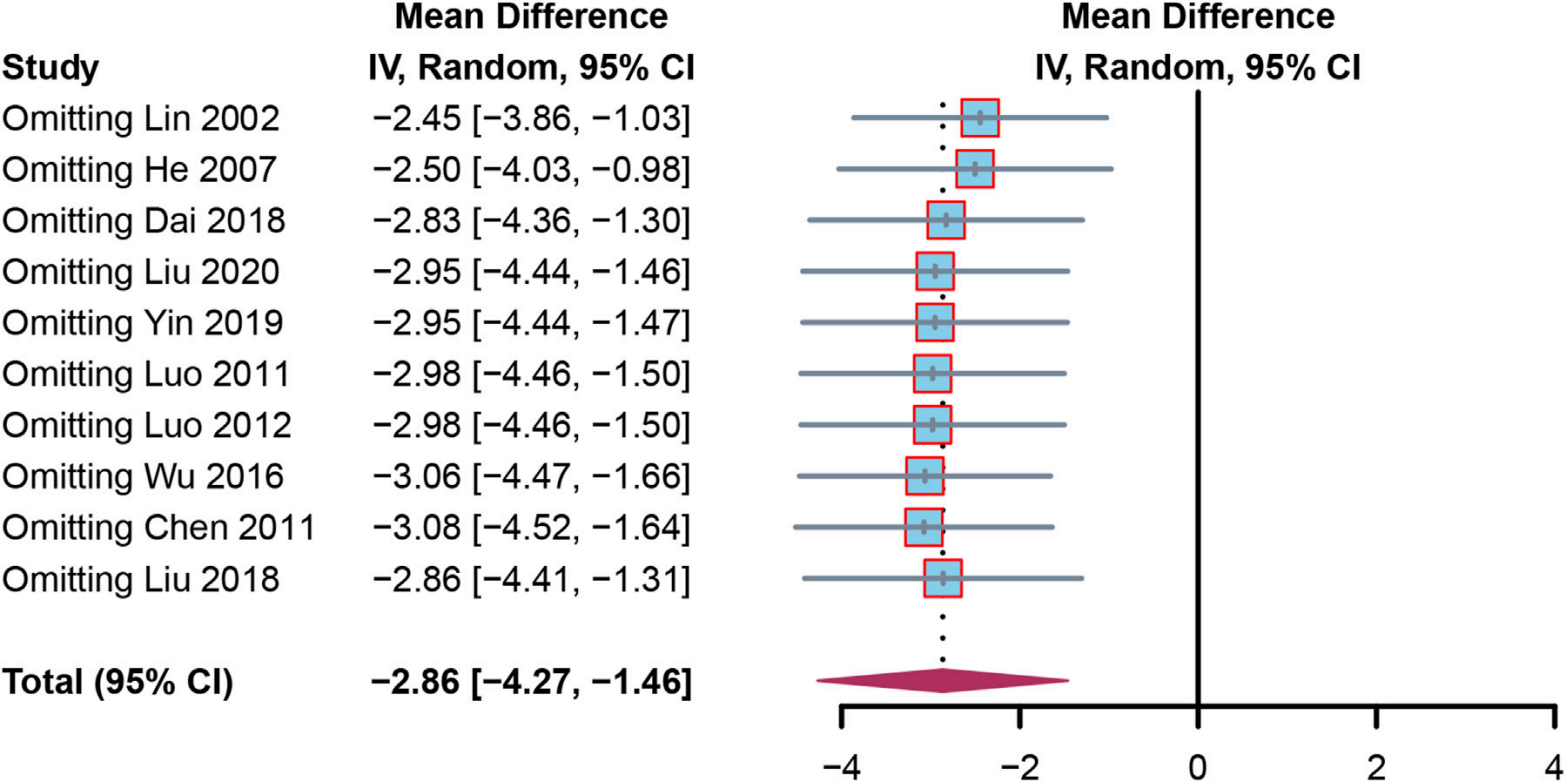
Sensitivity analysis by omitting each article in turn.

Sensitivity analysis was done to verify that the results were reliable. We removed each article in turn from the sensitivity forest plot (Figure 6). Each included article in the adverse events meta-analysis exhibited a comparable RR in terms of robustness, with Lin 2002 having the greatest RR at −2.45 [−3.86, −1.03] and Chen 2011 having the lowest RR at −3.08, 95%CI [−4.52, −1.64]. These findings suggested that the conclusions were sound.

## Discussion

The HAMD is a scale developed by Max Hamilton in 1960 to assess the symptoms of patients diagnosed with depressive states (Hamilton, 1960). Although the Patient Health Questionnaire-9 (PHQ-9) scale has been used more often in recent years to assess depressive states, the HAMD is still an accepted scale for the assessment of depression (Xiaonan Zhang, 2014; Zheng et al., 2020; Zhang et al., 2023). Using HAMD as an assessment outcome, the *Ginkgo biloba* extract group had lower HAMD values than the control group at 4 weeks, 6 weeks, and 8 weeks after taking the medication. The efficacy of adding *Ginkgo biloba* extract to the traditional treatment regimen for depression may be better than the traditional treatment regimen. In the results of other scales about stroke in patients with depression, the GKB group had a higher MBI than the control group. Also, the GKB group had lower MESSS scores than the control group. In addition to this, we also performed an analysis of serum biomarkers, including 5-HT and BDNF, and the results showed that the GKB group had higher levels of 5-HT and BDNF than the control groups. Again, these findings supported that the GKB groups had better CNS co-functional activity than patients treated with traditional depression medicine.

5-HT is a key excitatory neurotransmitter in the central nervous system that is found throughout the brain (Tao et al., 2023; Wu et al., 2023; Jinyi et al., 2024). Previous research has shown that 5-HT abnormalities are connected to the etiology of depression (Jaster et al., 2022; Jastrzebska et al., 2023). BDNF and 5-HT have a strong association in the typical human brain. 5-HT promotes BDNF production, and BDNF improves 5-HT signaling (Popova et al., 2022; Marcinkowska et al., 2023). However, this link is disturbed when the brain is ischemic and hypoxic, which may result in decreased production of BDNF and 5-HT (Kumar et al., 2018; Yadav et al., 2019; Kumar et al., 2020; Panigrahi et al., 2021; Mishra et al., 2022).

BDNF is broadly distributed throughout the brain, including the cerebral cortex and hippocampus (Kumar and Singh, 2016; Kumar and Singh, 2018; Kumar et al., 2022). It influences the release of neurotransmitters and trophic factors and encourages the differentiation and regeneration of injured neurons (Singh et al., 2023; Vyas et al., 2023). According to earlier research, BNDF is essential for preserving and fostering nerve fiber regeneration in monkey stroke models. Previous research revealed reduced serum BDNF levels in depressed people (Gabryelska et al., 2022; Gliwinska et al., 2023). According to a comprehensive study, antidepressant therapy may help depressed individuals’ serum BDNF levels rise (Kumar and Singh, 2015; Kumar and Sarveshanand, 2017; Kumar, 2021). Earlier research reported that BDNF plays a crucial role in the antidepressant effects of venlafaxine (Brammanathan et al., 2023).

Finally, the GKB group was also superior to the control group in terms of overall clinical efficacy. In the safety analysis results, no differences could be seen between the two groups. Regarding the comparative results in terms of adverse events, consistent with previous studies, there were no differences between the GKB and the control groups in terms of adverse events when treating depression and anxiety (Zhang et al., 2022). The clinical efficacy and safety of GKB on depression was consistent with previous research (Sarris et al., 2011).

We list various potential causes for the heterogeneity in some of our results. First, the variability in GKB dose may have an impact. Second, the different study sites of the collected publications unquestionably add to the variability. Third, there is a chance that personnel and research measurement variations will contribute to heterogeneity. Overall, this study suggested that GKB could lower the risk of depression or depressed symptoms based on a sizable sample and several indications. GKB was safe for patients with depression at the same time.

The current study had certain limitations. First, it was impossible to have a larger sample for each indication due to the variety of indicators reported in the included research. Second, comprehensive results for numerous populations were lacking because trials of GKB for patients with depression were not found in the United States or Europe. Last but not least, future indicators should contain more contemporary measurements like PHQ-9 and other biomarkers.

## Data Availability

The original contributions presented in the study are included in the article/Supplementary Material; further inquiries can be directed to the corresponding author.

## Abbreviations

GKB, *Ginkgo biloba*; PRISMA, Preferred Reporting Items for Systematic reviews and Meta-Analyses; INPLASY, International Platform of Registered Systematic Review and Meta-analysis Protocols; MD, mean difference; ORs, odds ratio; 95%CI, 95% confidence interval; BDNF, brain-derived neurotrophic factor; 5-HT, 5-hydroxytryptamine; HADS, Hospital anxiety depression Scale; MESSS, modified Edinburgh-Scandinavian Stroke Scale; PHQ-9, Patient Health Questionnaire-9; RCTs, randomized controlled trials; ROB 2.0, version 2 of the Cochrane risk-of-bias tool for randomized trials; HAMD, Hamilton Depression Scale.
