# Supplementary figures and images for: Effects and safety of Ginkgo biloba on depression: a systematic review and meta-analysis
Source: Front Pharmacol. 2024 Mar 18;15:1364030. doi: 10.3389/fphar.2024.1364030 (PMC10982363; doi:10.3389/fphar.2024.1364030)

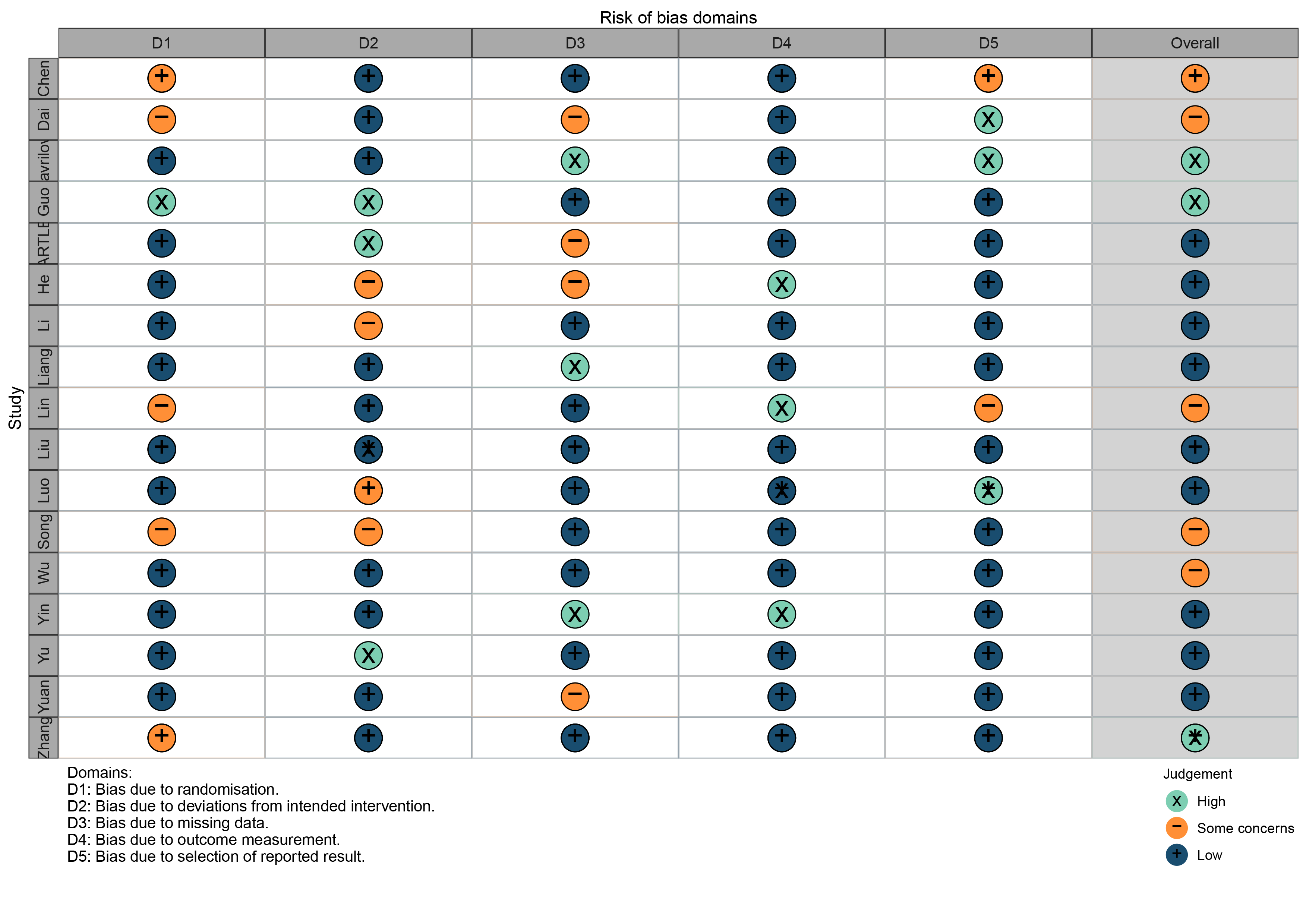

Supplement: Supplementary file 2 [file Image2.TIF]

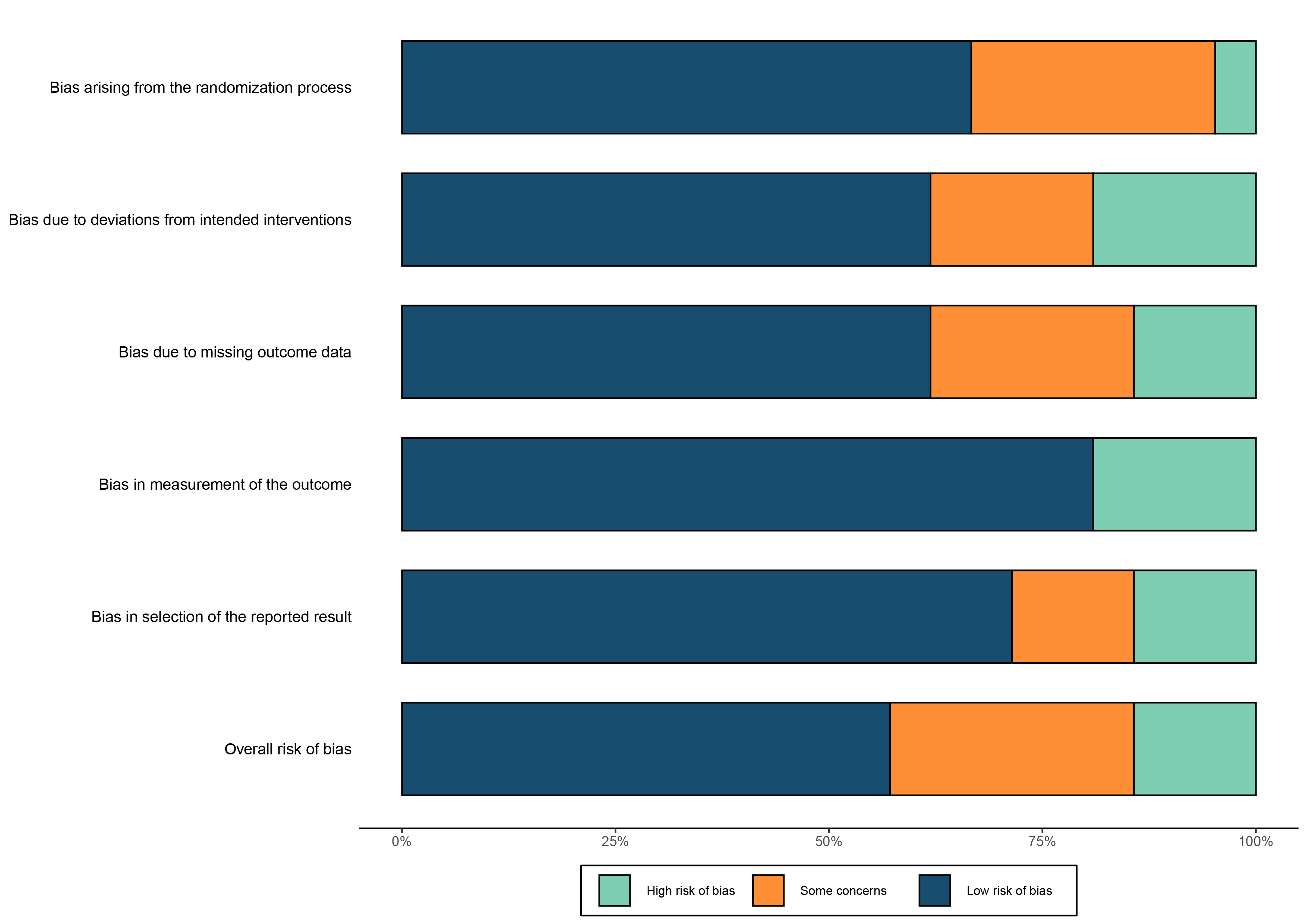

Supplement: Supplementary file 3 [file Image1.TIF]
